# Development of International Classification of Diseases crosswalks using text analysis methods.

**DOI:** 10.23889/ijpds.v9i5.2853

**Published:** 2024-09-18

**Authors:** Joykrishna Sarkar, Lisa Lix

**Affiliations:** 1 University of Manitoba

## Objectives

To evaluate the performance of a natural language processing (NLP) method to develop an automated crosswalk between the 9th and 10th revisions of the International Classification of Diseases (ICD) for diagnosis codes in the Charlson comorbidity index (CCI).

## Approach

SBERT, an advanced NLP transformer-based model, was used to produce sentence embeddings, numeric vectors that represent the semantic meaning of text, for the labels (i.e., descriptors) of 932 ICD-10-CA (Canadian Adaptation) codes in the CCI (up to six digits). Sentence embeddings were also produced for all ICD-9-CM (Clinical Modification) code labels (15,145). Cosine similarity scores (CSS) were calculated for all possible pairs of ICD-10-CA and ICD-9-CM code labels. CSSs were classified as equivalent (CSS = 1), high (0.8 ≤ CSS < 1), and low (CSS < 0.8). CSS categories for CCI diagnosis codes were compared to an ICD-9-CM to ICD-10-CA crosswalk file manually created by the Canadian Institute of Health Information.

## Results

Of the 932 CSSs for ICD-10-CA codes in CCI, 84 (9%) were classified as equivalent, 284 (30.5%) were high, and 564 (60.5%) were low. For ICD-10-CA codes with low CSSs, the median was 0.67 (interquartile range 0.14).

## Conclusions and Implications

An ICD-10-CA to ICD-9-CM crosswalk based on NLP had low accuracy for identifying semantically similar diagnosis code labels. The accuracy of this method might be improved by fine-tuning and training on task-specific data. Evaluation of different text analysis-based models would provide guidance for research involving ICD code labels.

